# Non-housekeeping genes expressed in human trabecular meshwork cell cultures

**Published:** 2012-01-28

**Authors:** Seyed Hassan Paylakhi, Shahin Yazdani, Craig April, Jian-Bing Fan, Hamidreza Moazzeni, Mostafa Ronaghi, Elahe Elahi

**Affiliations:** 1 School of Biology, Damghan University, Damghan, Iran; 2 Ophthalmic Research Center, Shahid Beheshti University of Medical Sciences, Tehran, Iran; 3 Illumina Inc, San Diego, CA; 4 Department of Cellular and Molecular Biology, International Campus, University of Tehran, Kish, Iran; 5 School of Biology, College of Science, University of Tehran, Tehran, Iran; 6 Department of Biotechnology, College of Science, University of Tehran, Tehran, Iran

## Abstract

**Purpose:**

To identify non-housekeeping genes definitively expressed in the human trabecular meshwork (TM).

**Methods:**

Microarray gene expression data on TM cultured cells from four studies were compared. Genes that were queried in at least three studies and assessed to be expressed in at least three studies were considered definitively expressed genes of the human TM. Housekeeping genes were removed from this set of genes. The non-housekeeping TM gene profile was analyzed for pathway enrichment and microRNA targeting, using bioinformatics tools. The results were compared with results of previous non-array based studies.

**Results:**

Nine hundred and sixty-two genes were identified as non-housekeeping TM expressed genes. Analysis of these by Kyoto Encyclopedia of Genes and Genomes led to identification of two enriched biologic pathways that achieved a highly significant Bonferroni p-value (p≤0.01): focal adhesion and extracellular matrix (ECM)-receptor interaction. Many of the genes were previously implicated in TM-related functions and the TM-associated disease glaucoma; however, some are novel. MicroRNAs known to be expressed in the trabecular meshwork were predicted to target some of the genes. Ten genes identified here, *ALDH1A1* (aldehyde dehydrogenase 1 family, member A1), *CDH11* (cadherin 11, type 2, OB-cadherin), *CXCR7* (chemokine (C-X-C motif) receptor 7), *CHI3L1* (chitinase 3-like 1), *FGF2* (fibroblast growth factor 2), *GNG11* (guanine nucleotide binding protein [G protein], gamma 11), *IGFBP5* (insulin-like growth factor binding protein 5), *PTPRM* (protein tyrosine phosphatase, receptor type, M), *RGS5* (regulator of G-protein signaling 5), and *TUSC3* (tumor suppressor candidate 3), were also reported as TM expressed genes in three earlier non-microarray based studies.

**Conclusions:**

A transcriptome consisting of 962 non-housekeeping genes definitively expressed in the human TM was identified. Multiple genes and microRNAs are proposed for further study for a better understanding of TM physiology.

## Introduction

The trabecular meshwork (TM) is located at the angle formed by the cornea and iris. The tissue is a major component of the conventional outflow pathway of aqueous humor and, thus, significantly modulates outflow of this fluid from the anterior chamber to venous blood via Schlemm’s canal [1]. Decreased outflow resulting from increased TM resistance causes increased intraocular pressure (IOP), which is a major risk factor for primary open angle glaucoma (POAG) [2-4]. The TM and aqueous humor also have roles in physiologic processes involving detoxification reactions and the immune system [5]. The precise mechanisms by which the TM affects these processes and regulates intraocular pressure are largely unknown. Analysis of the transcriptome of the TM and changes in the transcriptome due to conditions relevant to glaucoma may help illuminate these mechanisms. To this end, several studies have been performed within the past decade. Initially, human TM expressed genes were assessed by sequencing some clones of TM cDNA libraries [6,7]. Since the introduction of high-density chips for assessing gene expression profiles, four genome-wide TM transcriptome analyses that addressed glaucoma-relevant parameters and that included data on untreated TM cells have been published [8-11]. In one of the studies, gene expression profiles of cultured and native TM cells were compared, and it was reported that there was more than 90% similarity between expressed genes [10]. The remaining three studies used cultured TM cells. The data on the control cultures that were untreated in each of the four global studies allow assessment of the TM transcriptome. However, the profiles for the control cultures in these studies were not the same. The variations are due to a combination of factors, including individual variations, the complement of genes queried on the different chips used, and technical parameters of the hybridization reactions and analyses protocols. Here, we report results of a meta-analysis based on the four studies, with the objective of defining a TM transcriptome that is most likely to include only true positive non-housekeeping genes expressed in primary TM cultures of various sources. Housekeeping genes were designated by two recent studies on gene expression profiles of multiple human tissues using microarray and RNA deep sequencing data [12,13]. The derived non-housekeeping TM gene expression profile was analyzed using bioinformatics tools. The results were compared with those of previous studies on gene expression in normal human TM, including results of a recent study based on serial analysis of gene expression (SAGE) [6,7,14].

## Methods

Microarray gene expression data on control TM cultured cells from four studies were downloaded [8-11] (Gene Expression Omnibus [GEO] accession numbers GSE492, GSE4316, GSE7144, and GSE27275). Features of the microarray studies, including source of TM RNAs, chips used, and number of genes queried on the chips, are presented in Table 1. For the three studies in which Affymetrix chips were used, a hybridization threshold signal of 200 was used for expressed genes [8-10]. The same cutoff value was applied in the microarray study used here to define housekeeping genes; the cutoff was based on results of hybridizations to negative control probes placed on these chips and derived with the objective of achieving an optimal false positive to false negative ratio [12]. Others have also used the same cutoff [15]. Earlier studies based on instrument parameters and statistical analysis have recommended that signals lower than 200 on Affymetrix chips should not be considered in gene expression studies [16,17]. For the Illumina chips, a hybridization cutoff signal was chosen so that approximately the same fraction (50%) of genes queried on Affymetrix and Illumina chips would be considered genes expressed in the human TM. Hybridization signal of 200 was surpassed for almost all genes queried on the Illumina chips. Genes were grouped based on the number of studies in which they were queried. Subsequently, expressed genes were assessed with consideration of the number of studies in which hybridization thresholds were met and the number of studies in which they were queried. Only genes that were queried in at least three studies, and whose hybridization signals met the threshold in at least three studies, were considered definitively expressed genes of the human TM. Housekeeping (HK) genes were removed from this set of genes. Results of two recent studies that aimed to identify ubiquitously expressed genes in human cells were used to define housekeeping genes. One study, based on publically available microarray gene expression data on 18 human tissues, identified 2,403 genes expressed in at least 16 of those tissues [12]. The second study was more recent, and it was based on RNA deep sequencing data derived from diverse tissues and cell culture samples; it identified 7,897 ubiquitously expressed genes. Genes included in one or both of these studies (8,416 genes) were removed from the aforementioned list of definitively expressed genes of the human TM to derive a list of definitively expressed non-housekeeping genes of the human TM (non-HK TM genes). This set of genes was used in subsequent in silico analyses. Enriched functional pathways and functional categories or gene ontology terms annotated by Kyoto Encyclopedia of Genes and Genomes (KEGG) [18] and GO [19] were identified using The Database for Annotation, Visualization and Integrated Discovery (DAVID) bioinformatics resource [20]. A cutoff Bonferroni-p value of 0.01 was used for enriched KEGG pathways or GO functions. Non-HK TM genes whose expressions were altered by glaucoma-relevant experimental manipulations were identified. MicroRNAs whose target genes were enriched in the non-HK TM genes were also identified, using BioProfiling. Finally, non-HK TM genes identified here were compared to TM transcriptomes assessed by non-microarray protocols. The significance of the findings is discussed.

**Table 1:** Features of microarray studies used to assess gene expression in human trabecular meshwork.

**Reference of study**	**Source of data**	**Microarray chip**	No. genes queried*	No. genes expressed (%)**
[8]	Pooled RNA of 5 pairs of eyes; average of duplicate chips	Affymetrix Genome U95Av2Human	8963	4419 (49.3%)
[9]	Pooled RNA of 5 pairs of eyes; average of duplicate chips	Affymetrix Human Genome U133A	12962	7117 (54.9%)
[10]	RNA of 3 pairs of eyes; one chip per individual; average of 3 chips	Affymetrix Human Genome U133 plus 2.0	20203	10356 (51.2%)
[11]	RNA of 2 pairs of eyes; two chips per individual; average of 4 chips	Illumina HumanRf-8 V2	17133	8566 (50.0%)

* http://www.affymetrix.com/support/technical/libraryfilesmain.affx and http://www.illumina.com/support/annotation_files.ilmn. ** As per criteria described in text.

## Results

**Figure 1:**
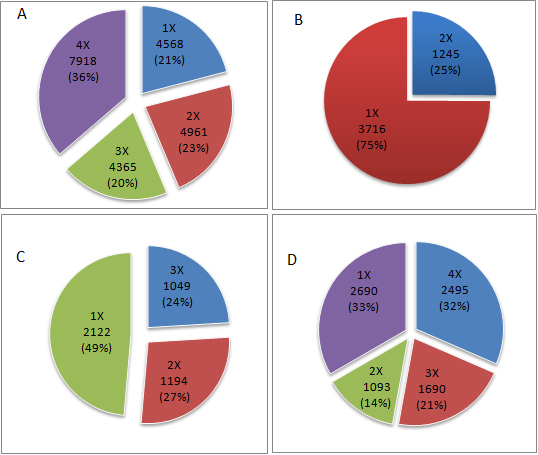
Number of genes expressed in human TM/number of times genes were queried on chips. **A**: Number of genes queried on chips of 1, 2, 3, or 4 of the microarray studies of this meta-analysis (% of all 21,813 genes queried at least once). **B**: Among genes queried 2×, number of genes assessed to be expressed in TM 1× or 2× (% genes queried 2×). **C**: Among genes queried 3×, number of genes assessed to be expressed in TM 1, 2, or 3× (% genes queried 3×). **D**: Among genes queried 4×, number of genes assessed to be expressed in TM 1, 2,3, or 4× (% genes queried 4×).

**Figure 2:**
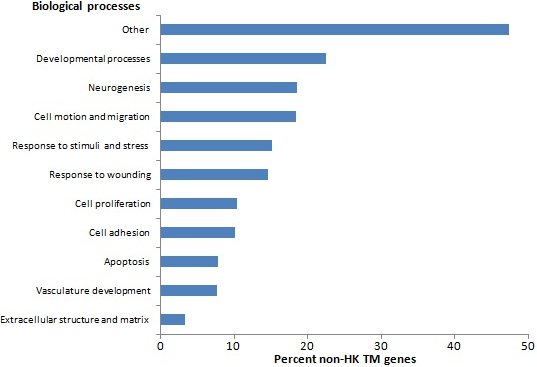
Percent of non-HK TM expressed genes enriched in related gene ontology biologic processes.

Approximately 50% of the genes queried on the chips of each study were expressed in the human TM (Table 1). A total of 21,813 genes were queried; the number of genes queried one, two, three, or four times are shown in Figure 1, and the genes are listed in Appendix 1. Of these, 5,379 genes met expression threshold criteria in at least three studies, and they are considered definitively expressed genes of the human TM. The majority of these (4,417 genes; 82%) were housekeeping genes; 962 (18%) were non-HK TM expressed genes (Appendix 1). Analysis of the 962 genes by KEGG led to identification of two enriched biologic pathways that achieved a highly significant Bonferroni p-value (p≤0.01): focal adhesion (Bonferroni p=0.00275) and extracellular matrix (ECM)-receptor interaction (Bonferroni p=0.00824). A third pathway, melanoma, achieved a significant Bonferroni p-value (p=0.01634). The non-HK TM genes involved in the focal adhesion and ECM-receptor interaction pathways are presented in Table 2. Analysis of the 962 genes by GO led to identification of many terms that achieved a highly significant Bonferroni p-value within the categories of biologic processes, molecular function, and cell component (Table 3). Related biologic processes were merged manually, on the basis of similarity between the processes and the fraction of non-HK TM genes associated with these processes, as shown in Figure 2. Genes whose expressions had been reported previously as changed due to glaucoma-relevant experimental manipulations were considered next [8-11]. The distribution of these genes among housekeeping and non-HK TM expressed genes is shown in Table 4, and the genes included among the non-HK genes are presented in supplementary Appendix 2. There was a trend toward preferential distribution of the genes within the non-HK TM class of genes in all four studies tested, although the difference reached statistical significance in only two of the studies. Notably, the preference is not observed in a study related to a non-TM ocular tissue [21] that was tested for comparison (Table 4). Subsequently, the 962 non-HK genes were submitted to BioProfiling to identify miRNAs that may preferentially target non-HK TM genes. No miRNA with a significant p-value was identified. However, enriched miRNA targets were identified for functionally clustered non-HK TM genes. Initially, the 962 non-HK TM genes were clustered into 26 groups by the Gene Functional Classification option within DAVID (Appendix 3). Eleven of the groups contained more than ten non-HK TM genes, and each of these groups was submitted separately to BioProfiling; ten is the default minimum number of genes accepted by this software. MicroRNAs with target gene enrichment (p≤0.05) were identified for four of the groups (Table 5).

**Table 2:** Biologic pathways enriched by KEGG analysis of non-housekeeping human TM expressed genes (Bonferroni p≤0.01).

**Pathway**	**Number of genes**	** Gene**
Focal adhesion (Bonferroni p=0.00275)	30	*CAV2, CAV1, TLN2, PIP5K1C, VTN, MYL9, COL6A3, COL6A2, PDGFC, COL11A1, THBS2, EGFR, COL4A2, COL4A1, PIK3CD, MET, ITGA2, HGF, FLNC, COL5A2, COL5A1, VEGFC, LAMA4, CCND2, ITGA5, ITGA7, PDGFRA, PDGFRB, COL1A1, MYLK*
ECM-receptor interaction (Bonferroni p=0.00824)	17	*COL4A2, COL4A1, HSPG2, ITGA2, VTN, COL5A2, COL5A1, LAMA4, SDC1, ITGA5, ITGA7, COL6A3, COL6A2, SV2A, COL1A1, THBS2, COL11A1*

**Table 3:** Gene ontology terms enriched by analysis of non-housekeeping human TM expressed genes (Bonferroni p≤0.01).

**Category**	**Term**	**Gene annotation**	**No. genes**	**Bonferroni p-value**
Biologic process	GO:0048731	System development	227	1.88E-18
Biologic process	GO:0032502	Developmental process	280	2.45E-18
Biologic process	GO:0048856	Anatomic structure development	239	4.89E-18
Biologic process	GO:0007275	Multicellular organismal development	259	2.44E-17
Biologic process	GO:0048513	Organ development	177	4.28E-15
Biologic process	GO:0009653	Anatomic structure morphogenesis	134	1.13E-13
Biologic process	GO:0042127	Regulation of cell proliferation	95	1.88E-10
Biologic process	GO:0032501	Multicellular organismal process	320	7.73E-10
Biologic process	GO:0030198	Extracellular matrix organization	28	1.08E-08
Biologic process	GO:0051270	Regulation of cell motion	38	2.41E-08
Biologic process	GO:0007399	Nervous system development	109	3.33E-07
Biologic process	GO:0042060	Wound healing	36	3.35E-07
Biologic process	GO:0040012	Regulation of locomotion	36	3.89E-07
Biologic process	GO:0042221	Response to chemical stimulus	122	5.17E-07
Biologic process	GO:0022603	Regulation of anatomic structure morphogenesis	38	1.10E-06
Biologic process	GO:0043062	Extracellular structure organization	32	1.52E-06
Biologic process	GO:0006928	Cell motion	60	2.88E-06
Biologic process	GO:0030334	Regulation of cell migration	32	3.86E-06
Biologic process	GO:0001944	Vasculature development	40	4.58E-06
Biologic process	GO:0032879	Regulation of localization	70	5.99E-06
Biologic process	GO:0009888	Tissue development	74	7.37E-06
Biologic process	GO:0001568	Blood vessel development	39	7.86E-06
Biologic process	GO:0050793	Regulation of developmental process	74	1.33E-05
Biologic process	GO:0030154	Cell differentiation	141	1.38E-05
Biologic process	GO:0048522	Positive regulation of cellular process	154	2.02E-05
Biologic process	GO:0009887	Organ morphogenesis	65	2.12E-05
Biologic process	GO:0040017	Positive regulation of locomotion	23	2.16E-05
Biologic process	GO:0051272	Positive regulation of cell motion	23	2.16E-05
Biologic process	GO:0051239	Regulation of multicellular organismal process	92	3.57E-05
Biologic process	GO:0008284	Positive regulation of cell proliferation	52	5.50E-05
Biologic process	GO:0048869	Cellular developmental process	143	6.15E-05
Biologic process	GO:0009611	Response to wounding	61	7.10E-05
Biologic process	GO:0006950	Response to stress	141	8.91E-05
Biologic process	GO:0022008	Neurogenesis	66	1.06E-04
Biologic process	GO:0048518	Positive regulation of biologic process	162	1.65E-04
Biologic process	GO:0009605	Response to external stimulus	88	1.99E-04
Biologic process	GO:0048514	Blood vessel morphogenesis	33	2.49E-04
Biologic process	GO:0007167	Enzyme linked receptor protein signaling pathway	44	4.24E-04
Biologic process	GO:0001501	Skeletal system development	42	4.53E-04
Biologic process	GO:0048699	Generation of neurons	61	4.77E-04
Biologic process	GO:0048519	Negative regulation of biologic process	146	4.87E-04
Biologic process	GO:0030335	Positive regulation of cell migration	20	5.44E-04
Biologic process	GO:0048523	Negative regulation of cellular process	136	5.95E-04
Biologic process	GO:0007155	Cell adhesion	71	6.80E-04
Biologic process	GO:0022610	Biologic adhesion	71	7.21E-04
Biologic process	GO:0007169	Transmembrane receptor protein tyrosine kinase signaling pathway	33	0.001
Biologic process	GO:0065008	Regulation of biologic quality	122	0.0015
Biologic process	GO:0016477	Cell migration	37	0.0018
Biologic process	GO:0008285	Negative regulation of cell proliferation	44	0.0019
Biologic process	GO:0032101	Regulation of response to external stimulus	26	0.0033
Biologic process	GO:0043067	Regulation of programmed cell death	76	0.0053
Biologic process	GO:0035295	Tube development	31	0.0063
Biologic process	GO:0010941	Regulation of cell death	76	0.0064
Biologic process	GO:0042981	Regulation of apoptosis	75	0.0074
Biologic process	GO:0051674	Localization of cell	38	0.0088
Biologic process	GO:0048870	Cell motility	38	0.0088
Cellular component	GO:0044421	Extracellular region part	109	7.78E-13
Cellular component	GO:0005576	Extracellular region	172	7.52E-10
Cellular component	GO:0031012	Extracellular matrix	51	7.34E-09
Cellular component	GO:0044459	Plasma membrane part	179	1.96E-08
Cellular component	GO:0031226	Intrinsic to plasma membrane	114	5.43E-08
Cellular component	GO:0005578	Proteinaceous extracellular matrix	47	6.44E-08
Cellular component	GO:0005887	Integral to plasma membrane	110	2.62E-07
Cellular component	GO:0005615	Extracellular space	70	1.51E-05
Cellular component	GO:0005886	Plasma membrane	257	2.57E-05
Cellular component	GO:0005581	Collagen	13	3.45E-05
Cellular component	GO:0044420	Extracellular matrix part	23	3.87E-05
Cellular component	GO:0005624	Membrane fraction	73	9.01E-04
Cellular component	GO:0000267	Cell fraction	89	0.0027
Cellular component	GO:0005626	Insoluble fraction	73	0.0032
Molecular function	GO:0005515	Protein binding	509	4.31E-06
Molecular function	GO:0005102	Receptor binding	86	4.26E-05
Molecular function	GO:0005201	Extracellular matrix structural constituent	19	4.31E-04
Molecular function	GO:0008201	Heparin binding	20	0.0015
Molecular function	GO:0008083	Growth factor activity	25	0.0038
Molecular function	GO:0005539	Glycosaminoglycan binding	23	0.004
Molecular function	GO:0030247	Polysaccharide binding	24	0.0059
Molecular function	GO:0001871	Pattern binding	24	0.0059
Molecular function	GO:0004714	Transmembrane receptor protein tyrosine kinase activity	15	0.0084

**Table 4:** Distribution of genes with altered expression due to experimental manipulations among housekeeping and non-HK TM expressed genes^*^.

**Experimental manipulation**	**Reference**	(No.) % HK TM genes affected by experimental manipulation*	(No.) % non- HK TM genes affected by experimental manipulation*	**Statistical significance**	**% non-HK/% HK TM genes affected by experimental manipulation**
Treatment with prostoglandin analogs	8	(25) 0.57	(8) 0.83	NS (p<0.50)	1.47
TGFβ^¥^ treatment	9	(35) 0.79	(49) 5.09	p<0.001	6.43
Glaucoma versus non-glaucoma TM cells	10	(6) 0.14	(5) 0.51	NS (p~0.10)	3.82
*PITX2* knockdown	11	(11) 0.25	(9) 0.94	p~0.001	3.76
Effect of aging on retina^&^	21	(8) 0.18	(1) 0.10	NS (p<0.75)	0.56

^*^Affected genes are as reported in respective studies, and HK and non-HK genes refer to genes identified in present study; ^¥^ transforming growth factor β; ^&^ represents a control treatment relating to a non-TM ocular tissue; NS=not significant.

**Table 5:** MicroRNAs with target enrichment in functionally grouped non-HK TM genes*.

**Group no.**	**miRNA with enriched targets**	**No. target genes (No. genes in group)**	**Target genes**	**p value**
3	HSA-MIR-29A.5	8 (10)	*COL1A1, COL4A1, COL4A2, COL4A5, COL5A1, COL5A2, COL7A1, COL11A1*	2.85E-09
3	HSA-MIR-29C.5	8 (10)	*COL1A1, COL4A1, COL4A2, COL4A5, COL5A1, COL5A2, COL7A1, COL11A1*	5.12E-09
3	HSA-MIR-29B.5	7 (10)	*COL4A1, COL4A2, COL4A5, COL5A1, COL5A2, COL7A1, COL11A1*	3.91E-07
3	HSA-MIR-29A.4	8 (10)	*COL1A1, COL4A1, COL4A2, COL4A5, COL5A1, COL5A2, COL7A1, COL11A1*	8.04E-07
3	HSA-MIR-29B.4	8 (10)	*COL1A1, COL4A1, COL4A2, COL4A5, COL5A1, COL5A2, COL7A1, COL11A1*	9.36E-07
3	HSA-MIR-29C.4	8 (10)	*COL1A1, COL4A1, COL4A2, COL4A5, COL5A1, COL5A2, COL7A1, COL11A1*	1.05E-06
3	HSA-LET-7A.4	6 (10)	*COL1A1, COL4A1, COL4A2, COL4A5, COL5A1, COL5A2*	0.0014
3	HSA-LET-7C.4	6 (10)	*COL1A1, COL4A1, COL4A2, COL4A5, COL5A1, COL5A2*	0.0015
3	HSA-LET-7B.4	6 (10)	*COL1A1, COL4A1, COL4A2, COL4A5, COL5A1, COL5A2*	0.0017
3	HSA-LET-7G.4	5 (10)	*COL1A1, COL4A1, COL4A2, COL4A5, COL5A2*	0.01
3	HSA-LET-7F.4	5 (10)	*COL1A1, COL4A1, COL4A2, COL4A5, COL5A2*	0.01
3	HSA-LET-7E.4	5 (10)	*COL1A1, COL4A1, COL4A2, COL4A5, COL5A2*	0.01
3	HSA-LET-7I.4	5 (10)	*COL1A1, COL4A1, COL4A2, COL4A5, COL5A2*	0.01
3	HSA-MIR-98.4	5 (10)	*COL1A1, COL4A1, COL4A2, COL4A5, COL5A2*	0.01
11	HSA-MIR-657.4	5 (13)	*FKTN, UST, GALNT10, XYLT1, GLT8D2*	0.01
13	HSA-MIR-517B.4	5 (49)	*SLC2A3, SCARA3, ORAI2, MBOAT2, C9ORF91*	0.01
13	HSA-MIR-188–3P.4	5 (49)	*SLC7A11, FAM119B, D4S234E, FAM171A1, FAM155A*	0.03
14	HSA-MIR-26A.5	6 (33)	*CDK6, DAPK1, PDGFRA, MAP3K2, STK39, TRIB2*	0.01
14	HSA-MIR-34A.5	5 (33)	*AXL, CDK6, EPHA4, MET, PDGFRA*	0.01
14	HSA-MIR-34C-5P.4	7 (33)	*AXL, CDK6, EPHA4, MET, PDGFRA, IKBKE, STK39*	0.01
14	HSA-MIR-34A.4	6 (33)	*AXL, CDK6, EPHA4, MET, PDGFRA, PRKD1*	0.01
14	HSA-MIR-26B.4	7 (33)	*CDK6, DAPK1, PDGFRA, WEE1, MAP3K2, STK39, TRIB2*	0.02
14	HSA-MIR-381.4	7 (33)	*EPHA4, GRK5, MET, RPS6KA3, TEK, WEE1, ZAK*	0.02
14	HSA-MIR-449B.4	5 (33)	*AXL, CDK6, EPHA4, PDGFRA, IKBKE*	0.02
14	HSA-MIR-26A.4	7 (33)	*CDK6, DAPK1, PDGFRA, WEE1, MAP3K2, STK39, TRIB2*	0.02
14	HSA-MIR-519C-5P.4	8 (33)	*AXL, CDK6, EPHA4, MYLK, PTK7, RPS6KA3, MAP3K2, ZAK*	0.02
14	HSA-MIR-26B.5	5 (33)	*CDK6, DAPK1, MAP3K2, STK39, TRIB2*	0.02
14	HSA-MIR-485–3P.4	5 (33)	*CDK6, DAPK1, MET, MELK, TRIB2*	0.03
14	HSA-MIR-323–3P.4	6 (33)	*DAPK1*, *MET*, *RPS6KA3*, *SGK1*, *MELK*, *MAP3K2*	0.03
14	HSA-MIR-32.4	6 (33)	*AXL*, *CDK6*, *SGK1*, *AATK*, *STK39*, *ZAK*	0.04
18	HSA-MIR-124.4	7 (19)	*AHR*, *E2F3*, *ELF4*, *ETS1*, *MITF*, *PLAGL2*, *SOX9*	0.01
18	HSA-MIR-145.4	5 (19)	*KLF5*, *ETS1*, *MAF*, *PLAGL2* SOX9	0.02
18	HSA-MIR-32.4	5 (19)	*E2F3*, *GATA2*, *MITF*, *SOX4*, *HAND1*	0.02

* miRNAs identified using BioProfiling under stringent setting; gene function groups identified by classification tool of DAVID.

Finally, the TM expressed genes identified here were compared to TM expressed genes reported earlier on the basis of sequencing in two TM cDNA libraries and one SAGE experiment [6,7,14]. As expected, most housekeeping genes commonly used as reference genes in expression studies, and expected to be expressed at high levels, were identified in all four studies (Table 6A). *HPRT1* (hypoxanthine phosphoribosyltransferase 1) was identified only in the microarray analysis presented here. Among the five known glaucoma-causing genes–* MYOC* (myocilin), *CYP1B1* (cytochrome P450, family 1, subfamily B, polypeptide 1), *OPTN* (optineurin), *WDR36* (WDR36), and *LTBP2* (latent transforming growth factor beta binding protein 2)–all except *WDR36* were identified in three of the studies (Table 6B) [22,23]. *WDR36* was not identified in any of the studies. *FOXC1* (forkhead box C1) and *PITX2* (paired-like homeodomain 2), genes associated with glaucoma-related Axenfeld-Rieger syndrome, were also identified in three of the studies [24]. The study in which the glaucoma-related genes were reported identified the lowest (386) number of TM expressed genes and, probably, only those highly expressed [6]. Only ten non-HK TM genes identified in the present analysis were identified in all four studies, and these are most likely to be highly expressed and consistently expressed non-housekeeping genes of the human TM (Table 7). The ten genes were *ALDH1A1* (aldehyde dehydrogenase 1 family, member A1), *CDH11* (cadherin 11, type 2, OB-cadherin), *CXCR7* (chemokine (C-X-C motif) receptor 7), *CHI3L1* (chitinase 3-like 1), *FGF2* (fibroblast growth factor 2), *GNG11* (guanine nucleotide binding protein (G protein), gamma 11), *IGFBP5* (insulin-like growth factor binding protein 5), *PTPRM* (protein tyrosine phosphatase, receptor type, M), *RGS5* (regulator of G-protein signaling 5), and *TUSC3* (tumor suppressor candidate 3). Among the interesting features of the ten genes is that five (*ALDH1A1*, *CDH11*, *CH13L1*, *FGF2*, and *IGFBP5*) were predicted by TargetScanHuman to be targeted by microRNA-23a and −23b cluster microRNAs (mir-23ab, mir-24, mir-27ab) and mir-204/211 (Table 7). Mir-24 and mir-204/211 are microRNAs experimentally confirmed to be expressed in the TM and to affect expression of some glaucoma-relevant genes [25,26]. Furthermore, the expression of mir-27, which was predicted to target *CH13L1* and *CXCR7,* may be regulated by the glaucoma-relevant transcription factor *PITX2* [27]. The ten genes identified in all the studies are further discussed in the discussion section below.

**Table 6:** TM expressed genes identified in microarray, cDNA library, and SAGE studies.

** **	**Study**				
** **	**Microarray studies^α^**		** **	** **	** **
**Gene**	**All genes**	**Non-HK genes**	**cDNA library 1^β^**	**cDNA library 2^γ^**	**SAGE^δ^**
**A. Commonly used housekeeping genes**					
*GAPDH*	+	-	+	+	+
*ACTB*	+	-	+	+	+
*B2M*	+	-	+	+	+
*HPRT1*	+	-	-	-	-
**B. Known glaucoma causing genes**					
*CYP1B1*	+	+	-	+	+
*MYOC*	+	+	-	+	+
*OPTN*	+	-	-	+	+
*WDR36*	-	-	-	-	-
*LTBP2*	+	-	-	+	+
*FOXC1*	+	+	-	-	+
*PITX2*	+	+	-	+	+

^α^ Present study; ^β^ reference [6]; ^γ^ reference [7]; ^δ^ reference [14]).

**Table 7:** Ten genes identified in all studies and miRNAs predicted to target these genes.

**Gene**	miRNAs*
*ALDH1A1*	mir-23ab
*CDH11*	mir-27ab, mir-144, mir-128, mir-26ab/1297, mir-214/761, mir-200bc/429, mir-101
*CXCR7*	mir-27ab, mir-142, mir-607, mir-524–5p, mir-520d-5p, mir-let7
*CHI3L1*	mir-24
*FGF2*	mir-23ab
*GNG11*	mir-587
*IGFBP5*	mir-24, mir-204/211, mir-130/301, mir-137, mir-139, mir-140, mir-193ab
*PTPRM*	mir-130/301, mir-34abc, mir-205, mir-218, mir-148, mir-152
*RGS5*	mir-182
*TUSC3*	mir-30abc

* Predicted by TargetScan.

## Discussion

The objective of this study was to identify non-housekeeping genes definitively expressed in human TM cultured cells. We aimed to minimize the consequences of individual variability and experimental techniques. To this end, we performed meta-analysis on four available microarray-based global gene expression studies on human TM cultured cells. The data was based on gene expression in 30 eyes of 15 individuals, 16–86 years of age. Cultured cells were used as surrogates for in vivo conditions, due to obvious limitations in procuring native tissue samples. Furthermore, as cultured cells are more likely to be used by others because of the same limitations, this analysis on cultured cells may be prove to be of greater use to other investigators. Greater than 90% similarity between transcriptomes of TM cultured and native cells has been reported, albeit the differences may be important [10]. Classification of housekeeping genes is somewhat arbitrary; we accepted all genes considered housekeeping in the most recent microarray and/or RNA deep sequencing based studies to be housekeeping genes. This low stringency was used to minimize the possibility of inclusion of housekeeping genes in our list of non-HK TM expressed genes. Ultimately, 962 TM expressed genes are proposed to be part of the non-housekeeping transcriptome of human TM cultured cells. Because stringent criteria were set for selection of these genes, they are highly likely to be TM expressed genes. Nevertheless, as only approximately half (4,417/8,416) of the genes considered here to be housekeeping genes met TM expression criteria, it is expected that the 962 non-HK genes are not inclusive of all non-HK genes expressed in the human TM. Indeed, a few genes whose expressions in the TM are strongly supported by experimental evidence were not among the 962 genes reported here, such as *MEIS2* (Meis homeobox 2) [11]. True non-HK genes that are not included among the 962 genes are likely biased toward those with lower levels of expression; they are unlikely to be biased with respect to function. The identified genes, therefore, are expected to reflect the morphological and functional properties of the human TM. They identify molecular markers to be addressed in studies on TM-related biology, including the TM-related disease glaucoma.

Analysis of the 962 genes by the DAVID bioinformatics tool identified focal adhesion and ECM-receptor KEGG-pathways to be enriched in the human TM at a highly significant Bonferroni p value level. A recently published gene expression profile of the normal human TM based on SAGE data also included many genes related to extracellular matrix, cell signaling, and cell structure/adhesion functions [14]. The extracellular matrix of the TM is important with respect to current understanding of TM function [28-31]. The importance of the ECM with respect to glaucoma is emphasized by the observation that two known glaucoma-causing genes are associated with this structure. The protein product of *MYOC*, the first identified POAG gene, interacts with components of the ECM [32,33]. Furthermore, the protein product of *ADAMTS10* (ADAM metallopeptidase with thrombospondin type 1 motif, 10), which has been identified as causative of glaucoma in a canine model of the disease, is involved in ECM formation [34,35]. Focal adhesions are specialized structures formed at contact points between cells and the ECM. In addition to structural links, some constituents of focal points are signaling molecules whose activities culminate in reorganization of the actin cytoskeleton that, in turn, affects cell shape, cell motility, and gene expression. Experimental evidence for involvement of focal adhesion proteins such as paxillin in signaling pathways in the human TM have been reported [36-38]. Among the 34 non-HK TM genes identified in the focal adhesion and ECM-receptor pathways, 17 were common to both; the overlap is to be expected, given the nature of the pathways (Table 2). Eight of the 17 common genes were collagen-coding genes. Common sequence variants near *CAV1* (caveolin 1) and *CAV2*, two of the genes only associated with focal adhesions, were recently reported to be associated with primary open-angle glaucoma [39]. The protein products of these genes affect IOP levels and are involved in nitric oxide and TGF-β (transforming growth factor beta) signaling and in the formation of caveolae [39]. Caveolae have been strongly implicated in signal transduction. *HGF* (hepatocyte growth factor), another of the focal adhesion associated genes, encodes hepatocyte growth factor. The concentration of this protein has been shown to be significantly higher in the aqueous fluid of glaucomatous eyes [40]. Furthermore, association between sequence variations in *HSP* and glaucoma has recently been reported in the Nepalese population [41]. Analysis of the 962 genes with DAVID also identified multiple enriched GO terms. ECM-related terms were enriched in all three term categories—biologic process, cellular component, and cellular function. Among the 14 cellular component-enriched terms, seven and five, respectively, were related to the ECM and plasma membrane. The genes associated with these terms constituted 400 of the 962 non-HK TM expressed genes. The high representation of ECM and plasma membrane-related terms is consistent with enriched focal adhesion and ECM-receptor KEGG pathways, and emphasizes that biologic processes related to these terms are integral to the biologic roles of the TM.

The glaucoma-relevant parameters that were tested in the genome-wide TM transcriptome analyses on which the present study is based, were treatments with prostaglandin analogs, transforming growth factor β, glaucoma status, and *PITX2* knockdown [8-11]. It was observed that these treatments preferentially affected the expression of genes classified here as non-HK TM expressed genes (Table 4). Although discussion of relevance of the genes with respect to the treatments is beyond the scope of this presentation, their enrichment in the non-HK class of genes suggests that the non-HK genes identified will be of value in studies aimed at achieving a better understanding of TM physiology.

Analysis that led to identification of miRNAs with enriched targets within one of the functionally related non-HK TM group of genes (group 3) proved interesting. All the genes placed within this group coded collagen proteins. Much evidence on the role of collagens in TM physiology and glaucoma pathogenesis is available in the literature [42-44]. Based on an association study, it was recently reported that the collagen-coding gene *COL5A1* (collagen, type V, alpha 1) affects central corneal thickness, which is a glaucoma risk factor [42]. Expressional elevation of *COL8A2* (collagen, type VIII, alpha 2) in TM cells in response to dexamethasone treatment has also been observed, and it has been suggested that this is part of the pathological process leading to steroid-induced glaucoma [45,46]. Notably, rare *COL8A2* mutations have been found in some glaucoma patients [47]. Results of experiments on cultured bovine TM cells have suggested that increased expression of collagen type 4 causes resistance to aqueous outflow [48]. Enriched target genes for all three members (miR-29a, b, and c) of the miR-29 family were found within group 3 genes. It is already known that miR-29 family members regulate expression of ECM proteins, including collagen types 1, 4, and 5, in human TM cultured cells [49] and in non-ocular tissues [50-55]. Experimental evidence for their effect on collagen type 7, which is predicted here, has not been reported. Effects of TGF-β and oxidative stress on ECM synthesis in the human TM may be partially mediated by miRNAs of this family [49]. Targets of six HSA-LET-7 family members were also significantly enriched in group 3 genes. To the best of our knowledge, experimental evidence for effects of these miRNAs on collagen expression is not available. HSA-LET-7F was also predicted to target *ELF4* (E74-like factor 4), *ETS1* (v-ets erythroblastosis virus E26 oncogene homolog 1), *PLAGL2* (pleiomorphic adenoma gene-like 2), *TEAD3* (TEA domain family member 3), and *HAND1* (heart and neural crest derivatives expressed 1) in group 18 genes, although the p value (p=0.06) in this case did not reach statistical significance (data not shown). Finally, numerous target genes for miR-26 and miR-34 family members were identified among group14 non-HK TM expressed genes.

In addition to the genes discussed above, some non-HK TM expressed genes identified in this study, as well as in previous TM transcriptome studies, are of particular interest. *CH13L1* has already been proposed as a candidate TM marker, due to its high expression in the TM and restricted expression in other tissues [10]. *CH13L1* codes a carbohydrate binding lectin. The protein directly interacts with type 1 collagen, and this interaction may affect tissue remodeling [56]. Involvement of *CH13L1* in the pathogenesis of glaucoma has been considered [57-59]. *ALDH1A1* codes an aldehyde dehydrogenase that may be involved in minimizing the deleterious effects of oxidative damage caused largely by exposure of the anterior chamber to ultraviolet radiation [11,60]. In addition, its expression in human TM cells is affected by TGF-β treatment [9]. TGF-β signaling and oxidative stress are both important components of glaucoma etiology [61]. *FGF-2* encodes basic fibroblast growth factor 2 and has roles in angiogenesis. Neovascular glaucoma is a rare form of glaucoma [62]. It has been shown that the hormone ghrelin inhibits FGF-2 mediated angiogenesis [63]. Notably, it has been reported recently that ghrelin levels are significantly lower in the aqueous humor of open-angle glaucoma patients, than in control individuals [64]. RGS*5* is of interest because an alternatively spliced form of its mRNA was identified in human ocular tissues, with highest expression of this form in the TM [65]. In addition, an *IGFBP-5* mRNA isoform with a unique 3′-untranslated region only expressed in the TM has been identified [66]. It would be of interest to determine if mir-24 and mir-204/211, predicted to target *IGFBP-5* mRNA, may selectively affect specific isoforms. It has already been shown that these miRNAs regulate multiple functions in TM [25,26]. To the best of our knowledge, TM-specific properties for CDH11, CXCR7, GNG11, PTPRM, and TUSC3 have not been reported. CDH11 is an adhesion molecule, and CXCR7, GNG11, and PTPRM are involved in cell signaling processes.

In conclusion, multiple protein coding genes and miRNAs are introduced here as candidates for study for better understanding of TM physiology. The observation that experimental data have already implicated many of the identified genes and miRNAs in ocular functions supports the premise that the remaining are also good candidates for further study.

## Appendix 1

Appendix 1. Four microarray/human TM gene expression studies: housekeeping and non-housekeeping genes expressed.****To access the data, click or select the words “Appendix 1.” This will initiate the download of a compressed (pdf) archive that contains the file.

## Appendix 2

Appendix 2. Non-HK TM genes with altered expression due to glaucoma-relevant exprimental manipulations. To access the data, click or select the words “Appendix 2.” This will initiate the download of a compressed (pdf) archive that contains the file.

## Appendix 3

Appendix 3. Non-HK human TM expressed genes grouped based on function. To access the data, click or select the words “Appendix 3.” This will initiate the download of a compressed (pdf) archive that contains the file.
